# *CircFOXO3* rs12196996, a polymorphism at the gene flanking intron, is associated with circFOXO3 levels and the risk of coronary artery disease

**DOI:** 10.18632/aging.103398

**Published:** 2020-07-02

**Authors:** Yu-Lan Zhou, Wei-Peng Wu, Jie Cheng, Li-Li Liang, Jin-Ming Cen, Can Chen, Xinguang Liu, Xing-Dong Xiong

**Affiliations:** 1 Institute of Aging Research, Guangdong Provincial Key Laboratory of Medical Molecular Diagnostics, Guangdong Medical University, Dongguan 523808, P.R. China; 2 Clinical Research Center, Affiliated Hospital of Guangdong Medical University, Zhanjiang 524001, P.R. China; 3 Department of Cardiovascular Disease, The First People’s Hospital of Foshan, Foshan 528000, P.R. China; 4 Department of Cardiovascular Disease, The Affiliated Hospital of Guangdong Medical University, Zhanjiang 524001, P.R. China; 5 Institute of Biochemistry and Molecular Biology, Guangdong Medical University, Zhanjiang 524001, P.R. China

**Keywords:** *circFOXO3*, single nucleotide polymorphism, rs12196996, coronary artery disease, risk

## Abstract

CircFOXO3 plays an important role in the pathogenesis of coronary artery disease (CAD). Single nucleotide polymorphisms (SNPs) at circRNA flanking introns may change its back-splicing and influence circRNA formation. Here, we aimed to investigate the influence of the polymorphisms at the *circFOXO3* flanking introns on individual susceptibility to CAD. A total of 1185 individuals were included in the case-control study. In a multivariate logistic regression analysis, we determined that the rs12196996 G variant was significantly associated with increased CAD risk (OR = 1.36, *P* = 0.014). A similar trend of the association was observed in the recessive model (OR = 2.57, *P* = 0.003). Stratified analysis revealed a more significant association with CAD risk among younger subjects and non-smokers. Consistent with these results, the haplotype rs12196996G-rs9398171C containing rs12196996G allele was also associated with increased CAD risk (OR = 1.31, *P* = 0.013). Further investigation revealed that the rs12196996 GG genotype was associated with decreased circFOXO3 expression, but not linear FOXO3 levels. Taken together, our data provide the first evidence that the rs12196996 polymorphism at the *circFOXO3* gene flanking intron is associated with CAD risk in the Chinese Han population, which is probably due to influence circFOXO3 levels.

## INTRODUCTION

Coronary artery disease (CAD) is the leading cause of morbidity and mortality worldwide, and its prevalence continues to increase. CAD is caused by stenosis of one of the coronary arteries due to plaque formation. When the stenosis is severe or a plaque ruptures, blood flow through the coronary artery is blocked, which causes myocardial infarction (MI) and sudden death [1]. Many risk factors reportedly contribute to the occurrence and development of CAD, including smoking, alcohol intake, diabetes, hypertension, hypercholesterolemia, obesity, physical inactivity, and the psychosocial situation [2]. Recently, accumulating studies have demonstrated the close associations of genetic variants or polymorphisms in candidate genes with CAD risk, providing evidence that host genetic variations exert critical roles on the pathogenesis of CAD, in addition to the above risk factors [3–5].

Circular RNAs (circRNAs) are a large group of transcripts that form covalently closed continuous loops [6]. They are expressed in a tissue-specific and developmental stage-specific manner [7]. CircRNAs regulate gene expression by acting as miRNA sponges, RNA-binding protein sequestering agents, or nuclear transcriptional regulators [8]. As a layer of the gene regulatory network, circRNA expression is an intermediate phenotype bridging genetic variants and phenotypic changes. Recent association studies have provided information on genetic factors, especially single nucleotide polymorphisms (SNPs), associated with variation in circRNA expression [9–11]. Interestingly, circRNA Quantitative Trait Loci (circQTL) SNPs were significantly enriched for the GWAS variants associated with various diseases [12]. Most circRNAs in humans are processed from internal exons with long flanking introns, usually containing inverted complementary sequences [13]. Liu et al*.* found that many circRNAs could be regulated by GWAS-linked circQTL SNPs located in flanking intron regions, which suggested the important roles of circRNA flanking introns in disease pathogenesis [12].

Circular RNA FOXO3 (CircFOXO3, also termed as hsa_circ_0006404) is derived from exon 3 of the forkhead box O3 (*FOXO3*) gene. A previous study demonstrated that circFOXO3 blocked cell cycle progression via forming ternary complexes with p21 and CDK2 [14]. Another study found that senescence-related proteins (ID-1 and E2F1) and stress-related proteins (FAK and HIF1a) could interact with circFOXO3 and were retained in the cytoplasm, resulting in increased cardiac senescence [15]. Xie et al*.* reported that the protective effect on the cardiovascular system by *Ganoderma lucidum* was through the regulation of circFOXO3 expression [16]. In addition, there are several binding sites for miR-149, miR-22, and miR-136 in circFOXO3, and these miRNAs are associated with CAD [17–20]. Thus, circFOXO3 plays an important role in the pathogenesis of CAD. Considering that genetic variations at the circRNA flanking introns can affect circRNA expression [9], we speculated that the polymorphisms at *circFOXO3* flanking introns could affect back-splicing, and in turn, the circFOXO3 expression, which consequently modulates an individual’s susceptibility to CAD. Therefore, we herein conducted a case-control study to elucidate the association of two tagSNPs at the *circFOXO3* flanking introns, namely rs12196996 and rs9398171, with the risk of CAD. We also investigated the association of the variants with expression levels of circFOXO3 in peripheral blood mononuclear cells (PBMCs) available from CAD patients and control subjects. Our results uncovered that the rs12196996 polymorphism at the *circFOXO3* gene flanking intron contributed to CAD risk in the Chinese Han population, which could be through influencing the expression levels of circFOXO3.

## RESULTS

### Characteristics of the study participants

We first performed a statistical power analysis using the PS program to verify whether the recruited samples could provide adequate power in identifying the association between the polymorphisms and CAD. Under the population parameter settings of the effect size of odd ratios of 1.36 and the allelic frequency of 0.161, our samples can provide a statistical power of 82.3% at the nominal Type I error rate of 0.05. The power analysis indicates that our sample size is sufficient for statistical analysis.

The baseline characteristics of CAD patients and controls are presented in Table 1. There was no statistically significant difference between cases and controls in terms of age. In comparison with control subjects, the CAD patients exhibited a higher proportion of male gender, smokers, and alcohol consumers. The clinical data on fasting plasma glucose, systolic, and diastolic blood pressure were found to be significantly elevated in the CAD group as compared with controls. In the lipid profiles comparison, TG and LDL-C were significantly higher in CAD patients than in controls, whereas serum HDL-C levels were significantly lower among CAD patients. In addition, patients with CAD were more likely to be diabetic, hypertensive, and dyslipidemia than the control subjects. In all, these data further demonstrated that male gender, smoking, alcohol intake, hypertension, diabetes, and hyperlipidemia were the important risk factors for developing CAD in the Chinese Han population.

**Table 1:** The characteristics of CAD cases and controls.

**Variable**	**Controls (n = 610)**	**Cases (n = 575)**	***P*^a^**
Age (years)	63.25 ± 11.11	64.18 ± 12.10	0.162
Sex (male)	369 (60.5%)	408 (71.0%)	<0.001
Smoking	139 (22.8%)	316 (55.0%)	<0.001
Drinking	65 (10. 7%)	136 (23.7%)	<0.001
Hypertension	232 (38.0%)	364 (63.3%)	<0.001
Diabetes	110 (18%)	278 (48.3%)	<0.001
Hyperlipidemia	223 (36.6%)	406 (70.6%)	<0.001
Systolic BP (mm Hg)	133.20 ± 19.18	140.11 ± 20.37	<0.001
Diastolic BP (mm Hg)	73.11 ± 10.52	75.49 ± 11.07	0.002
FPG (mM)	5.84 ± 1.93	6.67 ± 1.75	<0.001
TG (mM)	1.48 ± 0.86	2.08 ± 1.01	<0.001
TC (mM)	4.67 ± 1.12	4.67 ± 1.26	0.645
HDL-C (mM)	1.35 ± 0.39	1.20 ± 0.39	<0.001
LDL-C (mM)	2.69 ± 0.89	3.00 ± 0.92	<0.001

^a^ Two-sided chi-square test or independent-samples *t*-test.

### Multivariate associations of *circFOXO3* polymorphisms with the risk of CAD

Two tagSNPs (rs12196996 and rs9398171) located in *circFOXO3* flanking introns were genotyped in 575 CAD patients and 610 controls. The primary information for these variants is shown in Supplementary Table 1. The observed genotype frequencies of these variants were in Hardy-Weinberg equilibrium among the controls (all *P* values ≥ 0.05, Supplementary Table 1), providing no evidence of population stratification within the dataset. The multiple genetic models of *circFOXO3* tagSNPs and their associations with CAD risk are summarized in Table 2. From the allelic association analysis, we found that only rs12196996 showed statistical significance, and the G allele was associated with a significantly increased risk of CAD after adjustment for conventional risk factors (OR = 1.36, 95% CI = 1.06 - 1.73, *P* = 0.014). Further, the GG genotype exhibited an increased risk of CAD as well (OR = 2.36, 95% CI = 1.16-4.80, *P* = 0.018), compared to the AA genotype. We observed a similar association trend in the recessive model, the GG genotype was associated with increased CAD risk (OR = 2.21, 95% CI = 1.09-4.47, *P* = 0.027). Taken together, our data indicated that *circFOXO3* rs12196996 was associated with the CAD risk and that individuals carrying the G allele may have significantly increased CAD susceptibility. However, no significant association between rs9398171 and CAD risk was observed under the allelic and established genetic models (Table 2). MI is a primary manifestation of CAD. We also analyzed the association between the two tagSNPs and MI risk, and found that neither rs12196996 nor rs9398171 was associated with the MI risk in this study (Supplementary Table 2).

**Table 2:** Multivariate associations of tagSNPs at *circFOXO3* flanking introns with CAD risk.

**Models**		**Controls (n = 610)**	**Cases (n = 575)**	**OR (95% CI)**	***P*-value**	**OR (95% CI) ^a^**	***P*^a^**
		**No. (%)**	**No. (%)**				
***rs12196996***					
Allele	A	1023 (83.9)	918 (79.8)	1.00	-	1.00	-
	G	197 (16.1)	232 (20.2)	1.29 (1.05-1.59)	0.014	1.36 (1.06-1.73)	0.014
Genotype	AA	428 (70.1)	378(65.7)	1.00	-	1.00	-
	AG	167 (27.4)	162 (28.2)	1.10 (0.85-1.42)	0.474	1.24 (0.91-1.69)	0.166
	GG	15 (2.5)	35 (6.1)	2.64 (1.42-4.91)	0.002	2.36 (1.16-4.80)	0.018
Dominant	AA	428 (70.2)	378 (65.7)	1.00	-	1.00	-
	GG+AG	182 (29.8)	197 (34.3)	1.23 (0.96-1.57)	0.103	1.35 (1.00-1.80)	0.045
Recessive	AA+AG	595 (97.5)	540 (93.9)	1.00	-	1.00	-
	GG	15 (2.5)	35 (6.1)	2.57 (1.39-4.76)	0.003	2.21 (1.09-4.47)	0.027
***rs9398171***						
Allele	T	851 (69.8)	779 (67.7)	1.00	-	1.00	-
	C	369 (30.2)	371 (32.3)	1.10 (0.92-1.29)	0.305	1.12 (0.92-1.37)	0.265
Genotype	TT	301 (49.3)	276 (48.0)	1.00	-	1.00	-
	TC	249 (40.8)	227 (39.5)	1.31 (0.90-1.91)	0.165	1.05 (0.78-1.39)	0.766
	CC	60 (9.9)	72 (12.5)	0.99 (0.78-1.27)	0.963	1.35 (0.86-2.14)	0.193
Dominant	TT	301 (49.3)	276 (48.0)	1.00	-	1.00	-
	CT+CC	309 (50.7)	299 (52.0)	1.06 (0.84-1.33)	0.644	1.10 (0.84-1.45)	0.478
Recessive	CT+TT	550 (90.2)	503 (87.5)	1.00	-	1.00	-
	CC	60 (9.8)	72 (12.5)	1.31 (0.91-1.89)	0.143	1.33 (0.86-2.06)	0.204

^a^ Adjusted for age, sex, smoking, drinking, hypertension, diabetes, hyperlipidemia.

### Stratification analyses of *circFOXO3* rs12196996 with the risk of CAD

We further evaluated the alleles and CAD susceptibility stratified by age, gender and status of smoking and drinking (Table 3). The increased risk of CAD was more evident among younger subjects (≤ 60 years old, OR = 1.82, 95% CI = 1.19-2.76, *P* = 0.005) and non-smoker subjects (OR = 1.37, 95% CI = 1.02-1.85, *P* = 0.039) carrying the G allele. No further evident associations between rs12196996 alleles and CAD risk were observed among subgroups by gender or drinking.

**Table 3:** Multivariate associations of *circFOXO3* rs12196996 polymorphism with CAD risk by further stratification analysis.

**Variable**	**Allele**	**Controls No. (%)**	**Cases No. (%)**	**OR (95% CI)**	***P***
**Age ^a^**					
Age≤60	A	430 (85.7)	368 (80.0)	1.00	-
	G	72 (14.3)	92 (20.0)	1.82 (1.19-2.76)	0.005
Age>60	A	593 (82.6)	550 (79.7)	1.00	-
	G	125 (17.4)	140 (20.3)	1.16 (0.85-1.57)	0.350
**Gender^b^**					
Male	A	616 (83.5)	652 (79.9)	1.00	-
	G	122 (16.5)	164 (20.1)	1.26 (0.93-1.71)	0.142
Female	A	407 (84.4)	266 (79.6)	1.00	-
	G	75 (15.6)	68 (20.4)	1.49 (0.98-2.26)	0.062
**Smoking^c^**					
Yes	A	240 (86.3)	510 (80.7)	1.00	-
	G	38 (13.7)	122 (19.3)	1.30 (0.85-1.98)	0.226
No	A	783 (83.1)	408 (78.8)	1.00	-
	G	159 (16.9)	110 (21.2)	1.37 (1.02-1.85)	0.039
**Drinking^d^**					
Yes	A	109 (83.8)	215 (79.0)	1.00	-
	G	21 (16.2)	57 (21.0)	1.11 (0.57-2.19)	0.755
No	A	914 (83.9)	703 (80.1)	1.00	-
	G	176 (16.1)	175 (19.9)	1.35 (1.04-1.76)	0.026

^a^ Adjusted for gender, smoking, drinking, hypertension, diabetes, hyperlipidemia.

^b^ Adjusted for age, smoking, drinking, hypertension, diabetes, hyperlipidemia.

^c^ Adjusted for age, gender, drinking, hypertension, diabetes, hyperlipidemia.

^d^ Adjusted for age, gender, smoking, hypertension, diabetes, hyperlipidemia.

### Haplotype analysis of *circFOXO3* polymorphisms and the risk of CAD

**Figure 1:**
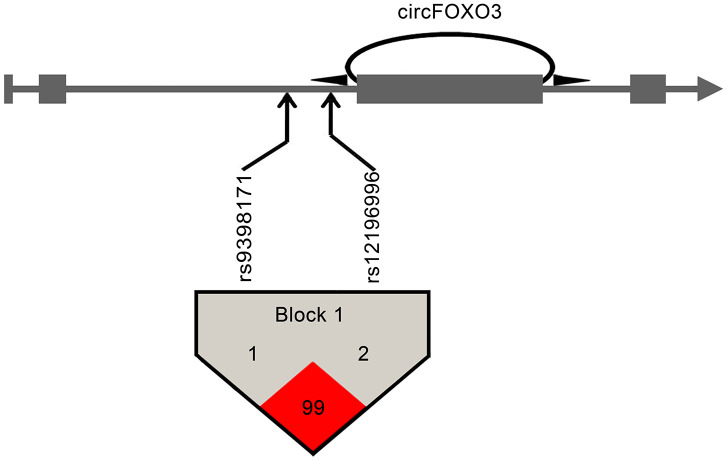
**Pairwise linkage disequilibrium between *circFOXO3* variants.** CircFOXO3 derived from the third exon of the FOXO3 gene. Arrows indicate the locations of the SNPs. Linkage disequilibrium analysis revealed that the two tagSNPs at *circFOXO3* flanking introns were located in the same haplotypic block. Numbers within squares indicate the D' value reported as a percentile.

Linkage disequilibrium (LD) analysis for the two tagSNPs was performed using the Haploview platform [21]. As shown in Figure 1, the two tagSNPs (rs12196996 and rs9398171) were in linkage disequilibrium (D’ = 0.99), indicating that they were located in one haplotypic block. The frequencies of derived common haplotypes (>3%) and their risk prediction for CAD are summarized in Table 4. The haplotype rs12196996G - rs9398171C carrying the G allele of rs12196996 was found to be associated with increased risk of CAD (OR = 1.31, 95% CI = 1.06-1.61, *P* = 0.013). For further stratified analysis, this haplotype appeared to increase risk of CAD in younger subjects and non-smokers (Table 4).

**Table 4:** Haplotype analysis of tagSNPs at *circFOXO3* flanking introns and CAD risk.

**Haplotype^a^**	**Controls**	**Cases**	**OR (95% CI)**	***P***
	**No. (%)**	**No. (%)**		
**Total**	n = 610	n = 575		
rs12196996A-rs9398171C	172.01 (14.1)	142.12 (12.2)	0.85 (0.67-1.08)	0.171
rs12196996A-rs9398171T	850.99 (69.8)	777.88 (67.6)	0.91 (0.76-1.08)	0.283
rs12196996G-rs9398171C	196.99 (16.1)	230.88 (20.1)	1.31 (1.06-1.61)	0.013^\^
**Age ≤ 60**	n = 251	n = 230		
rs12196996A-rs9398171C	68.00 (13.5)	55.00 (12.0)	0.87 (0.59-1.27)	0.461
rs12196996A-rs9398171T	362.00 (72.1)	313.00 (68.0)	0.82 (0.62-1.09)	0.168
rs12196996G-rs9398171C	72.00 (14.3)	92.00 (20.0)	1.49 (1.06-2.10)	0.020
**Non-smokers**	n = 471	n = 259		
rs12196996A-rs9398171C	131.00 (13.9)	53.00 (10.2)	0.71 (0.50-0.99)	0.043
rs12196996A-rs9398171T	652.00 (69.2)	355.00 (68.5)	0.97 (0.77-1.22)	0.788
rs12196996G-rs9398171C	159.00 (16.9)	110.00 (21.2)	1.33 (1.01-1.74)	0.040

^a^Haplotypes with frequency less than 3% were excluded.

### Association of rs12196996 with the expression of *circFOXO3*

**Figure 2:**
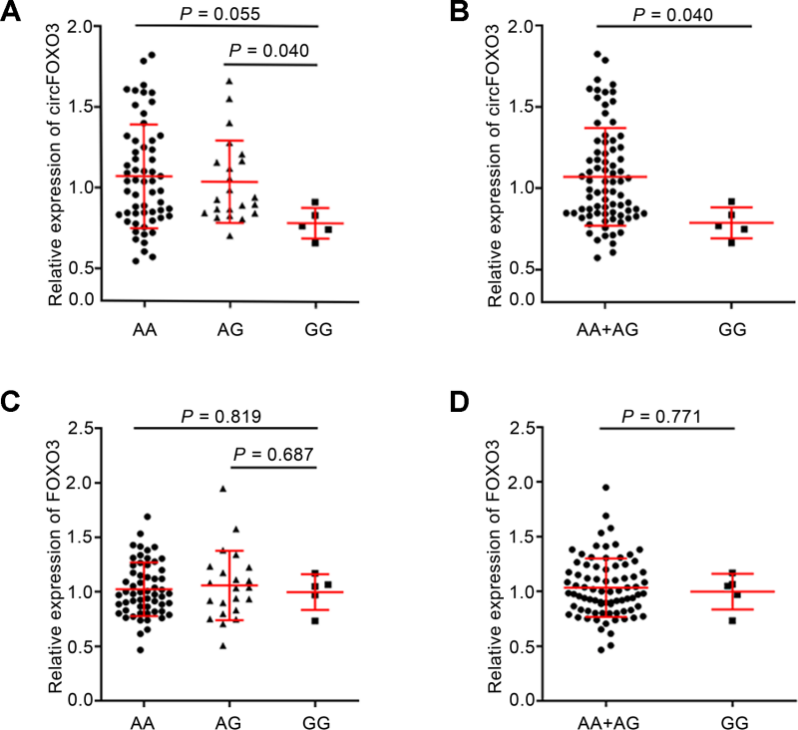
**Association between rs12196996 and the expression levels of circFOXO3 or linear FOXO3 in PBMCs in the sum of CAD patients and control subjects.** Analysis of circFOXO3 expression levels in PBMCs of individuals carrying AA vs. AG vs. GG genotypes (**A**) or the combined AA+AG genotypes vs. GG genotype (**B**). Analysis of linear FOXO3 expression levels in PBMCs of individuals carrying AA vs. AG vs. GG genotypes (**C**) or the combined AA+AG genotypes vs. GG genotype (**D**).

To further investigate the functional relevance of the *circFOXO3* rs12196996 polymorphism, we conducted a correlation analysis between the genotypes and the expression levels of circFOXO3 or linear FOXO3 using real-time quantitative RT-PCR. Direct sequencing of circFOXO3 PCR products confirmed the presence of the back-spliced exons 3, joined by a head-to-tail splice junction (Supplementary Figure 1). As shown in Figure 2A, the expression level of circFOXO3 decreased in subjects carrying the GG genotype than in those with the AA or AG genotypes. Similarly, a significant association between the GG genotype and lower levels of circFOXO3 was observed when compared with the combined AA+AG genotypes (*P* = 0.040, Figure 2B). However, there was no significant association between rs12196996 and the expression level of linear FOXO3 (Figure 2C and 2D).

## DISCUSSION

CircRNAs are abundant in eukaryotic transcriptomes and have been linked to various human disorders [15, 22, 23]. Most of them are processed from internal exons with long flanking introns [24]. A recent study reported that genetic variants located in flanking sequences more likely contributed to circRNA biogenesis, and were highly linked to genome-wide association study signals of complex diseases [12]. In this study, we studied two tagSNPs (rs12196996 and rs9398171) located in *circFOXO3* flanking introns and found that rs12196996 was associated with the risk of CAD, and the increased risk was more evident among younger subjects and non-smokers carrying the G allele. Haplotype rs12196996G-rs9398171C containing the rs12196996 G allele also conferred susceptibility to CAD in the Chinese Han population. Furthermore, we observed that rs12196996 was associated with circFOXO3 expression, but not linear FOXO3 expression.

Several lines of evidence indicated that circRNAs were aberrantly expressed in several vascular diseases, neurological disorders, and cancers [25–27]. Unlike linear RNAs, circRNAs are impacted less by technical and biological effects, mainly because circRNAs are more stable than linear RNAs. However, genetic factors, i.e., circQTLs, may contribute to circRNA expression variation. Recent association studies showed that the expression level of specific circRNAs may be influenced by the genotype of disease-associated SNPs [10, 11, 28]. For example, circular ANRIL was significantly decreased in individuals harboring the risk (G) allele of rs10757278, which was associated with atherosclerosis [11]. The CAD-protective haplotype at chromosome 9p21 locus, which consisted of rs10757274, rs2383206, rs2383207, and rs10757278, have significantly increased expression of circular ANRIL [28]. In addition, a circRNA derived from a multiple sclerosis (MS)-associated locus, hsa_circ_0043813 from the STAT3 gene, can be modulated by the three genotypes at the disease-associated SNP [10]. These data suggested that the expression of circRNA could be regulated by polymorphisms in the circRNA gene.

Polymorphisms within flanking introns of circRNA play important roles in circRNA expression and pathogenesis [9, 11, 12]. A study from Burd et al*.* revealed that rs7341786, within 200 bp of an ANRIL intron-exon boundary, could promote the production of cANRIL [11]. Additionally, Liu et al*.* reported that a subset of circQTL SNPs, located in flanking introns, could regulate circRNA expression, which was highly linked to genome-wide association study signals of complex diseases [12]. Ahmed et al. identified thousands of circRNAs from RNAseq data and observed an enrichment of the circQTL variants at the proximity of the back-splice junction. Furthermore, these circQTLs are associated with circRNA abundance and exist independently of expression quantitative trait loci (eQTLs) with most circQTLs exerting no effect on mRNA expression [9]. In this study, we found that CAD-associated SNP rs12196996 at the *circFOXO3* flanking intron could influence circFOXO3 expression rather than linear FOXO3 expression, suggesting that rs12196996 might influence circFOXO3 formation, and then modulate the individual’s susceptibility to CAD.

In the stratified analysis, our data revealed that the increased risk of the rs12196996 G allele in CAD was more remarkable amongst younger subjects (≤60 years old) in allelic or haplotypic analyses, while no significant association was observed in the older group (*>*60 years old). These results are in agreement with other studies reporting the differential effects of age on the association of gene polymorphisms with cardiovascular diseases [29–31]. The potential explanation to this phenomenon was that the dominant cause of CAD pathogenesis in older subjects is more likely due to aging effects (e.g., weak immune system, relative high-level exposure to environmental risk factors) rather than direct genetic effects. Previous studies have reported associations between smoking and CAD [32]. In this study, the association between the rs12196996 polymorphism and CAD risk was more pronounced in non-smokers. Cigarette smoke contains a number of oxidizing compounds and is an important source of free radicals, which contributes to both the development of atherosclerosis and increases the incidence of cardiovascular events [33, 34]. The differences observed for smoking may mask the influence of individual variants of this polymorphism in the present study population.

Several limitations should to be addressed in this study. First, the cases and controls were enrolled from hospitals and may not represent the general population. Nonetheless, the genotype distribution of the control subjects was in Hardy-Weinberg equilibrium. Second, the sample size of the present study was not large enough, especially for subgroup analyses. Finally, given that the results of this study were not replicated, further studies in different populations should be employed to validate the significance of the association between these polymorphisms and CAD risk.

In summary, our study provides the first evidence that the rs12196996 polymorphism at *circFOXO3* flanking intron, which links to aberrant circFOXO3 expression, is associated with CAD risk, suggesting that this polymorphism may be employed as a biomarker in assessing the risk of developing CAD. Clearly, further studies with a larger sample size and in diverse ethnic populations are necessary to confirm the general validity of our findings.

## MATERIALS AND METHODS

### Study subjects

In this case-control study, a total of 1185 Chinese Han subjects with 575 CAD patients and 610 controls were consecutively recruited from the First People’s Hospital of Foshan (Foshan, China) and the Affiliated Hospital of Guangdong Medical University (Zhanjiang, China) between March 2011 and October 2015. The patients were recruited from the Cardiology Department of the participating hospitals. All patients were newly diagnosed and previously untreated. CAD was defined as angiographic evidence of at least one segment of a major epicardial coronary artery with more than 50% organic stenosis. The diagnosis of MI was based on clinical symptoms and typical electrocardiographic changes, and on increases in serum cardiac markers, such as creatinine kinase, aspartate aminotransferase, lactate dehydrogenase, and troponin T. The diagnosis was further confirmed by the identification of the responsible stenosis in any of the major coronary arteries or in the left main trunk by coronary angiography. Control subjects were also recruited from the two hospitals for regular physical examinations during the same period when CAD patients were recruited. Individuals with congestive heart failure, peripheral vascular disease, rheumatic heart disease, pulmonary heart disease, chronic kidney, hepatic disease, or any malignancy were excluded from the study.

All enrolled subjects were genetically unrelated Han Chinese. Each subject was interviewed after written informed consent was obtained, and a structured questionnaire was administered by interviewers at the enrollment to collect information on demographic data and risk factors related to CAD. The diagnosis of hypertension was established if patients were on antihypertensive medication or if the mean of three measurements of systolic blood pressure (SBP) ≥ 140 mm Hg or diastolic blood pressure (DBP) ≥ 90 mm Hg were obtained. Diabetes mellitus was defined as fasting blood glucose ≥ 7.0 mmol/L or use of antidiabetic drug therapy. Dyslipidemia was defined as serum total cholesterol (TC) concentration > 5.72 mmol/L or triglyceride (TG) concentration > 1.70 mmol/L or use of lipid-lowering therapy. Smokers were defined as individuals who had smoked once a day for over one year. Drinkers were defined as those who consumed ≥ 30 g of alcohol/week on average for at least one year. The study was approved by the Medical Ethics Committee of the above two hospitals, and written consent was obtained before the commencement of the study.

### DNA extraction

Two to three ml of peripheral whole blood was collected from each study participant into tubes containing EDTA (BD Vacutainers, Franklin Lakes, USA). All samples were immediately stored at -80 °C. Genomic DNA was isolated from peripheral whole blood using the TIANamp blood DNA extraction kit (TianGen Biotech, Beijing, China) according to the manufacturer’s instructions. All DNA samples were stored at -80 °C until use.

### TagSNP selection and genotyping

Many single nucleotide polymorphisms (SNPs) show correlated genotypes, or linkage disequilibrium (LD), suggesting that only a subset of SNPs (known as tagging SNPs, or tagSNPs) are required to be genotyped for disease association studies [35]. In this study, the whole *circFOXO3* (hsa_circ_0006404) sequence and its flanking intron sequences were scanned for tagSNPs. Polymorphisms were selected on the basis of the 1000 Genomes Project database (https://www.internationalgenome.org/1000-genomes-browsers). The Haploview software (version 4.2) was a prerequisite for tagSNP selection with minor allele frequency (MAF) larger than 0.05, and LD patterns with *r*^2^ *>* 0.8 [36]. Totally, two tagSNPs (rs12196996 and rs9398171) at the *circFOXO3* flanking introns were selected for genotyping. The positions of the two tagSNPs are shown in Figure 1. The haplotypic blocks of the two tagSNPs were estimated using the Haploview software. The haplotype analysis was performed using SHEsis software (http://analysis.bio-x.cn/myAnalysis.php) [37].

Genomic DNA was genotyped by the PCR-ligase detection reaction (PCR-LDR) method as described previously [38]. The sequences of primers and probes used for PCR-LDR are listed in Supplementary Table 3. In order to verify the accuracy of the data, 10% of samples were genotyped in duplicate to check for concordance and the results were 100% concordant.

### RNA extraction and real-time quantitative RT-PCR

Total RNA was extracted from peripheral blood mononuclear cells (PBMCs) of 85 individuals using Trizol (Invitrogen, Carlsbad, USA) according to the manufacturer’s instructions. The quality and quantity of the RNAs were assessed by A260/A280 nm reading using a Nanodrop 2000 spectrophotometer (Thermo Fisher Scientific, USA). The RNA integrity was determined by running an aliquot of the RNA samples on a denaturing agarose gel electrophoresis. SYBR green-based quantitative real-time polymerase chain reaction with a divergent primer couple was used to detect the expression levels of circFOXO3 and linear FOXO3. The sequences of primers and probes are listed in Supplementary Table 4. Results were normalized to the expression levels of the ACTIN housekeeping gene, and three technical replicates were performed for each sample.

### Statistical analysis

The statistical power analysis was performed using PS program (Power and Sample size calculations, Version 3.0.43). Hardy-Weinberg equilibrium was tested using a goodness-of-fit χ2 test on the controls. Data were presented as mean ± S.D. for the quantitative variables and percentages for the qualitative variables. Differences regrading demographic and clinical characteristics between cases and controls were estimated using the Student’s *t*-test (for continuous variables) and χ2 test (for categorical variables). Association between the polymorphisms and CAD risk was evaluated using logistic regression analysis, adjusted by age, sex, smoking, drinking, hypertension, diabetes, and hyperlipidemia. The statistical analyses were performed using SPSS version 21.0. Statistical differences of circFOXO3/linear FOXO3 expression levels among different groups in real-time quantitative RT-PCR experiments were determined by Mann-Whitney U-test. *P* < 0.05 was considered statistically significant for all tests.

## Supplementary Material

Supplementary Figure 1

Supplementary Tables
