# Supplementary material for: CircFOXO3 rs12196996, a polymorphism at the gene flanking intron, is associated with circFOXO3 levels and the risk of coronary artery disease
Source: Aging (Albany NY). 2020 Jul 2;12(13):13076–89. doi: 10.18632/aging.103398 (PMC7377899; doi:10.18632/aging.103398)
Supplement: Supplementary Figure 1 [file aging-12-103398-s001..pdf]

SUPPLEMENTARY FIGURE

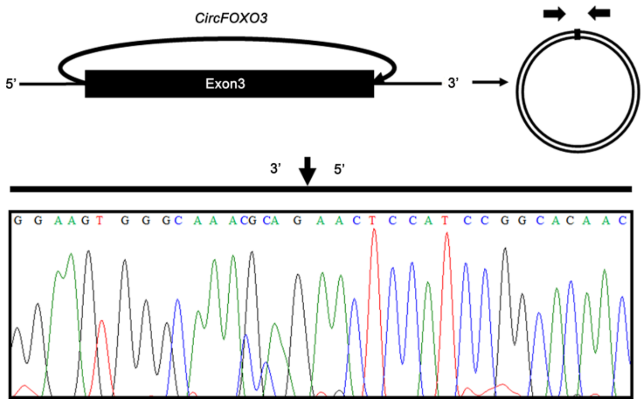

Supplementary Figure 1. Schematic representation of formation of the circFOXO3 through a back-splicing event at exon 3. The curved arrow joins the 5' splice site to the 3' splice site of exon 3. On the right, a schematic representation of circFOXO3 is depicted; arrows indicate the divergent primer couple used to detect circFOXO3. Below the scheme, direct-sequencing electropherogram shows the head-to-tail splice junction, indicated by a black arrow, located between the 3' and 5' splice sites of exon 3.
