# Supplementary material for: CircFOXO3 rs12196996, a polymorphism at the gene flanking intron, is associated with circFOXO3 levels and the risk of coronary artery disease
Source: Aging (Albany NY). 2020 Jul 2;12(13):13076–89. doi: 10.18632/aging.103398 (PMC7377899; doi:10.18632/aging.103398)
Supplement: Supplementary Tables [file aging-12-103398-s002..pdf]

SUPPLEMENTARY TABLES

Supplementary Table 1. Primary information for polymorphisms at *circFOXO3* flanking introns.

Genotyped SNPs	rs12196996	rs9398171
ChrPos (Genome Build 108)	108984067	108983527
Pos in *circFOXO3*gene[a]	-590bp	-1130bp
MAF for Chinese[b] (CHB and CHS) in HapMap[a]	0.127	0.240
MAF in our controls (n = 610)	0.161	0.302
P-Value for HWE test in our controls[c]	0.781	0.431

[a] Distance to backsplice junction of the *circFOXO3*.
[b] MAF: minor allele frequency.
[c] HWE: Hardy-Weinberg equilibrium.

Supplementary Table 2. Multivariate associations of tagSNPs at *circFOXO3* flanking introns with MI risk.

Models		Controls (n = 610) No. (%)	Cases (n = 283) No. (%)	OR (95% CI)[a]	*P*-value[a]
rs12196996					
Additive	A	1023 (83.9)	455 (80.4)	1.00	-
	G	197 (16.1)	111 (19.6)	1.35 (1.00-1.84)	0.052
Genotype	AA	428 (70.2)	186 (65.7)	1.00	-
	AG	167 (27.4)	83 (29.4)	1.32 (0.90-1.93)	0.153
	GG	15 (2.5)	14 (4.9)	1.96 (0.82-4.69)	0.130
Dominant	AA	428 (70.2)	186 (65.7)	1.00	-
	AG+GG	182 (29.8)	97 (34.3)	1.39 (0.96-1.99)	0.078
Recessive	AG+AA	595 (97.5)	269 (95.1)	1.00	-
	GG	15 (2.5)	14 (4.9)	1.81 (0.76-4.28)	0.178
rs9398171					
Additive	T	851 (69.8)	387 (68.4)	1.00	-
	C	369 (30.2)	179 (31.6)	1.09 (0.84-1.40)	0.520
Genotype	TT	301 (49.3)	136 (48.1)	1.00	-
	TC	249 (40.8)	115 (40.6)	1.20 (0.68-2.13)	0.524
	CC	60 (9.8)	32 (11.3)	1.07 (0.75-1.52)	0.726
Dominant	TT	301 (49.3)	136 (48.1)	1.00	-
	CT+CC	309 (50.7)	147 (51.9)	1.09 (0.78-1.53)	0.606
Recessive	CT+TT	550 (90.2)	251 (88.7)	1.00	-
	CC	60 (9.8)	32 (11.3)	1.17 (0.68-2.02)	0.575

[a] Adjusted for age, sex, smoking, drinking, hypertension, diabetes, hyperlipidemia.

Supplementary Table 3. The sequences of primers and probes used to genotype *circFOXO3* polymorphisms.

Name	Sequence (5'-3')
**Primers**	
rs12196996-F	AGTGTGAACTTCAATATGGGC
rs12196996-R	GCTGGGAATAGATAAGCTCAC
rs9398171-F	TGGAAGGCAGACCACAGAAG
rs9398171-R	GCAACTTTAGAGTGGAGAAAC
**Probes**	
rs12196996-FAM	P-TGCCCATATTGAAAGGCCCCTTTTTTTTTTTTTTTTTT-FAM
rs12196996-A	TTTTTTTTTTTTTTTTTCCTGGCCTTTCTGTAATTATAT
rs12196996-G	TTTTTTTTTTTTTTTTTTTCCTGGCCTTTCTGTAATTATAC
rs9398171-FAM	P-GTCAATATCTACAAGGATAATTTTTTTTTTTTTTTTTTTTTTTTTTTT-FAM
rs9398171-C	TTTTTTTTTTTTTTTTTTTTTTTTTTCACTATTTCAGTAGGTGATCGGG
rs9398171-T	TTTTTTTTTTTTTTTTTTTTTTTTTTTTCACTATTTCAGTAGGTGATCGGA

Supplementary Table 4. Primers used for quantitative real-time PCR (qRT-PCR).

Name	Sequence (5'-3')
ACTIN	F: AGATGACCCAGATCATGTTTGAG
	R: AGGGCATACCCCTCGTAGAT
circ*FOXO3*	F: TTTGATTCCCTCATCTCCACA
	R: GAGTTCTGCTTTGCCCACTT
FOXO3	F: TGGCAAGCACAGAGTTGGATGAAG
	R:CATATCAGTCAGCCGTGGCAGTTC
